# Genomic modulators of the immune response

**DOI:** 10.1016/j.tig.2012.10.006

**Published:** 2013-02

**Authors:** Julian C. Knight

**Affiliations:** Wellcome Trust Centre for Human Genetics, University of Oxford, Roosevelt Drive, Oxford, OX3 7BN, UK

**Keywords:** immunity, major histocompatibility complex, gene regulation, autoimmunity, immunodeficiency, leukocyte

## Abstract

Our understanding of immunity has historically been informed by studying heritable mutations in both the adaptive and innate immune responses, including primary immunodeficiency and autoimmune diseases. Recent advances achieved through the application of genomic and epigenomic approaches are reshaping the study of immune dysfunction and opening up new avenues for therapeutic interventions. Moreover, applying genomic techniques to resolve functionally important genetic variation between individuals is providing new insights into immune function in health. This review describes progress in the study of rare variants and primary immunodeficiency diseases arising from whole-exome sequencing (WES), and discusses the application, success, and challenges of applying genome-wide association studies (GWAS) to disorders of immune function and how they may inform more rational use of therapeutics. In addition, the application of expression quantitative-trait mapping to immune phenotypes, progress in understanding MHC disease associations, and insights into epigenetic mechanisms at the interface of immunity and the environment are reviewed.

## Genetic variation and the immune system: in health and disease

**Figure 1:**
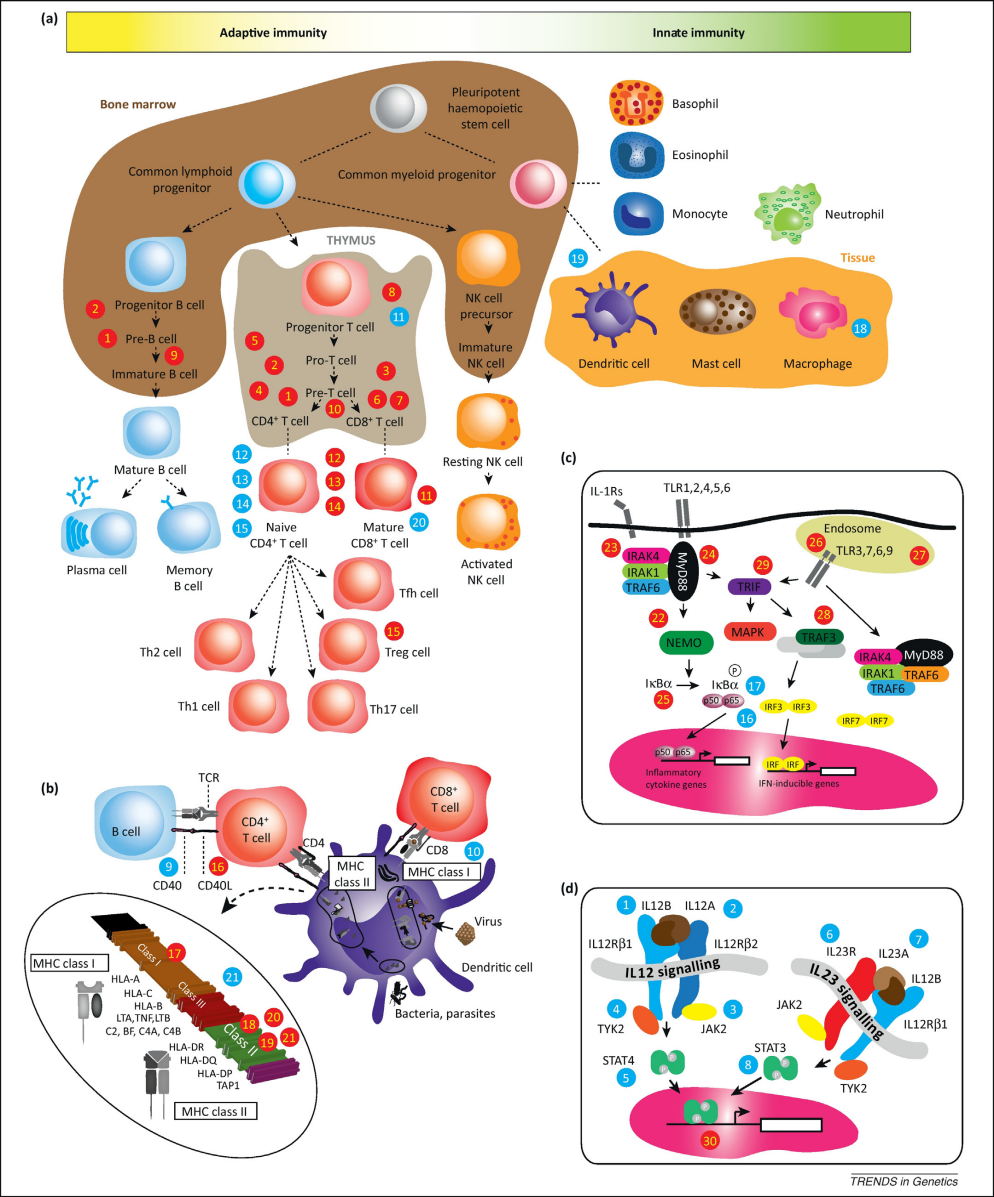
Overview of key mediators of innate and adaptive immunity, development, and signalling. This is presented to provide context for genetic variants implicated in different Mendelian diseases with immune phenotypes (indicated by red filled circles numbered from 1 to 30), and common autoimmune diseases (blue filled circles numbered from 1 to 21). These numbers correspond to the diseases described in Figures 2 and 3. **(a)** Development of the myeloid and lymphoid lineages is shown together with key cell types involved in innate and adaptive immunity. **(b)** The role of an antigen-presenting cell (illustrated here for a dendritic cell) is shown, including antigen presentation via the endogenous pathway (MHC class I molecules) or the exogenous pathway (MHC class II molecules). The MHC gene locus is shown in terms of the classical class I and class II regions, with the intervening class III region. **(c)** Toll-like receptor signalling is a key component of the innate immune response involved in pathogen recognition (Box 1). **(d)** IL-12/IL-23 receptors and the JAK–STAT signalling pathways are central to the cytokine cascade and inflammatory response together with modulation/expansion of Th17 cells. Genetic variation in genes encoding different proteins in these pathways has been associated with autoimmunity by recent GWAS implicating dysregulation of IL-12 signalling (Th1 cells) and IL-23 signalling (Th17 cells) [6]. Abbreviations: IKK, inhibitor of κ light polypeptide gene enhancer in B cells kinase; IL-1Rs, interleukin-1 receptors; IRAK, IL-1R-associated kinase; IRF, interferon regulatory factor; JAK, Janus kinase; MHC, major histocompatibility complex; MyD88, myeloid differentiation primary-response protein 88; NEMO, NF-κB essential modulator; NK cell, natural killer cell; STAT, signal transducer and activator of transcription; TCR, T cell receptor; Tfh, follicular helper T cell; Th, T helper cell; TLR, Toll-like receptor; TRAF, TNF receptor-associated factor; TRAM, TRIF-related adaptor molecules; Treg, regulatory T cell; TYK, tyrosine kinase.

**Figure 2:**
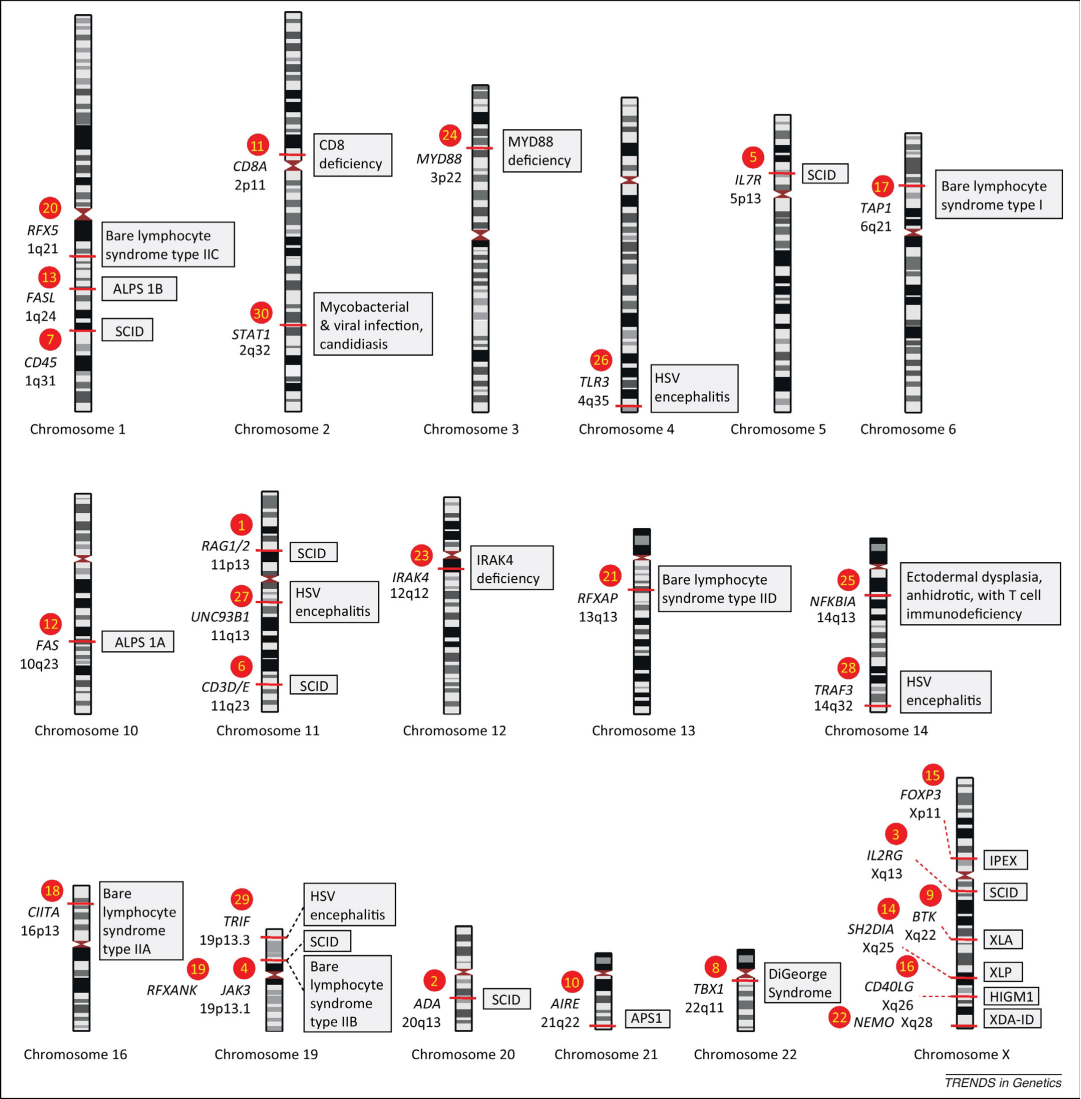
Examples of Mendelian traits involving immune defects. Red filled numbered circles correspond to those shown in the overview of the immune system (Figure 1). The implicated gene (given in italics) and chromosomal position are shown together with the associated phenotype (grey shaded box). Note that the genetic variants involved range from point mutations (single-nucleotide variants) to large structural variants. Full descriptions of all traits are available at the Online Mendelian Inheritance in Man (OMIM) database (http://omim.org/). For the severe combined immunodeficiency (SCID) syndromes shown (numbered circles 1–7) these can be classified into T cell negative, B cell negative, NK cell negative type (T^–^B^–^NK^–^) (OMIM #102700) (circle 2); T^–^B^–^NK^+^ (OMIM #601457) (circle 1); T^–^B^+^NK^–^ (OMIM #300400, #600802, #601457) (circles 3,4,7); T^–^B^+^NK^+^ (OMIM #608971) (circles 5,6). DiGeorge syndrome (OMIM #188400) (circle 8) due to deletion at chr22q11 includes a T cell deficit due to thymic hypoplasia. X-linked agammaglobulinemia (XLA) (OMIM #300755) (circle 9) due to mutation involving the *BTK* gene is an immunodeficiency syndrome characterised by failure to produce mature B cells and of Ig heavy-chain rearrangement. Autoimmune polyendocrine syndrome (APS) (OMIM #240300) (circle 10) due to mutations in *AIRE* is characterised by two of three of Addison disease (adrenal insufficiency), hypoparathyroidism, and chronic mucocutaneous candidiasis, and arises due to failure of central immune tolerance. CD8 deficiency (OMIM #608957) (circle 11) is characterised by the absence of CD8^+^ T cells. Autoimmune lymphoproliferative syndrome (ALPS) (OMIM #601859) manifests with autoreactive lymphocytes due to disordered apoptosis, either ALPS type 1A (circle 12) due to mutation in the *FAS* gene, or ALPS type 1B (circle 13) involving the FAS ligand (*FASL*) gene. X-linked lymphoproliferative syndrome (XLP) (OMIM #308240), due to mutation in *SH2D1A* (circle 14), results in severe immunodysregulation, notably in the context of viral infection. IPEX (immunodysregulation, polyendocrinopathy, and enteropathy X-linked syndrome) (OMIM #304790) (circle 15) is an X-linked disorder associated with severe diarrhoea, T1D, and dermatitis due to mutation in *FOXP3*. Hyper IgM syndrome 1 (HIGM1) (OMIM #308230) (circle 16) due to mutation in *CD40LG*. Bare lymphocyte syndrome type I (OMIM #604571) (circle 17) involves failure of expression of HLA class I genes due to mutation in *TAP1*, *TAP2*, or *TABP* genes, and has a relatively mild phenotype with chronic bacterial infections. By contrast, bare lymphocyte syndrome type II (OMIM ##209920) is associated with severe combined immunodeficiency with different complementation groups, group A (mutation in *CIITA*) (circle 18), group B (mutation in *RFXANK*) (circle 19), group C (mutation in *RFX5*) (circle 20), and group D (mutation in *RFXAP*) (circle 21). Mutations involving TLR signalling [85] are illustrated (numbered circles 24–29). Mutations involving the canonical pathway include X-linked recessive anhidrotic ectodermal dysplasia with immunodeficiency (XDA-ID) (OMIM #300291) due to hypomorphic mutations in *NEMO* (circle 22), a critical subunit of the inhibitory IKK complex, resulting in defective NF-κB signalling and susceptibility to infection; IRAK4 deficiency (OMIM #607676) (circle 23) and MYD88 deficiency (OMIM #612260) (circle 24), involving genes encoding adaptors recruited during TLR signalling in response to microbial products, resulting in autosomal recessive conditions and pyogenic bacterial infections; and ectodermal dysplasia, anhidrotic, with T cell immunodeficiency (OMIM #164008) (circle 25) due to mutation in *NFKB1A* and altered IκBα activity. Mutations in the alternative TLR pathway, and that are associated with susceptibility to viral infections such as herpes simplex virus (HSV) encephalitis, include *TLR3* (OMIM #613002) (circle 26), *UNC93B* (OMIM #610551) (circle 27), *TRAF3* (circle 28) and *TRIF* (circle 29).

Effective immune function is critical to health, and dysregulation underlies a large proportion of human diseases. Elucidation of the genetic basis of rare heritable diseases involving deficiencies in the innate and adaptive immune response have been highly informative in advancing our understanding of both disease mechanisms and of the fundamental processes underpinning immunity (Box 1, Figures 1 and 2) [1–3]. Current genomic technologies, primarily driven by high-throughput genotyping and DNA sequencing, are revolutionising our ability to resolve those remaining rare diseases which have not proved tractable using classical genetic approaches and to address meaningfully the heritable basis of common diseases in which immune dysregulation plays a significant role [4,5]. Moreover, we now have the tools to interrogate normal variation between healthy individuals to assess genetic modulators of immune function and determine the extent of context-specific effects on immune function dependent on cell or tissue type and environmental factors.

**Figure 3:**
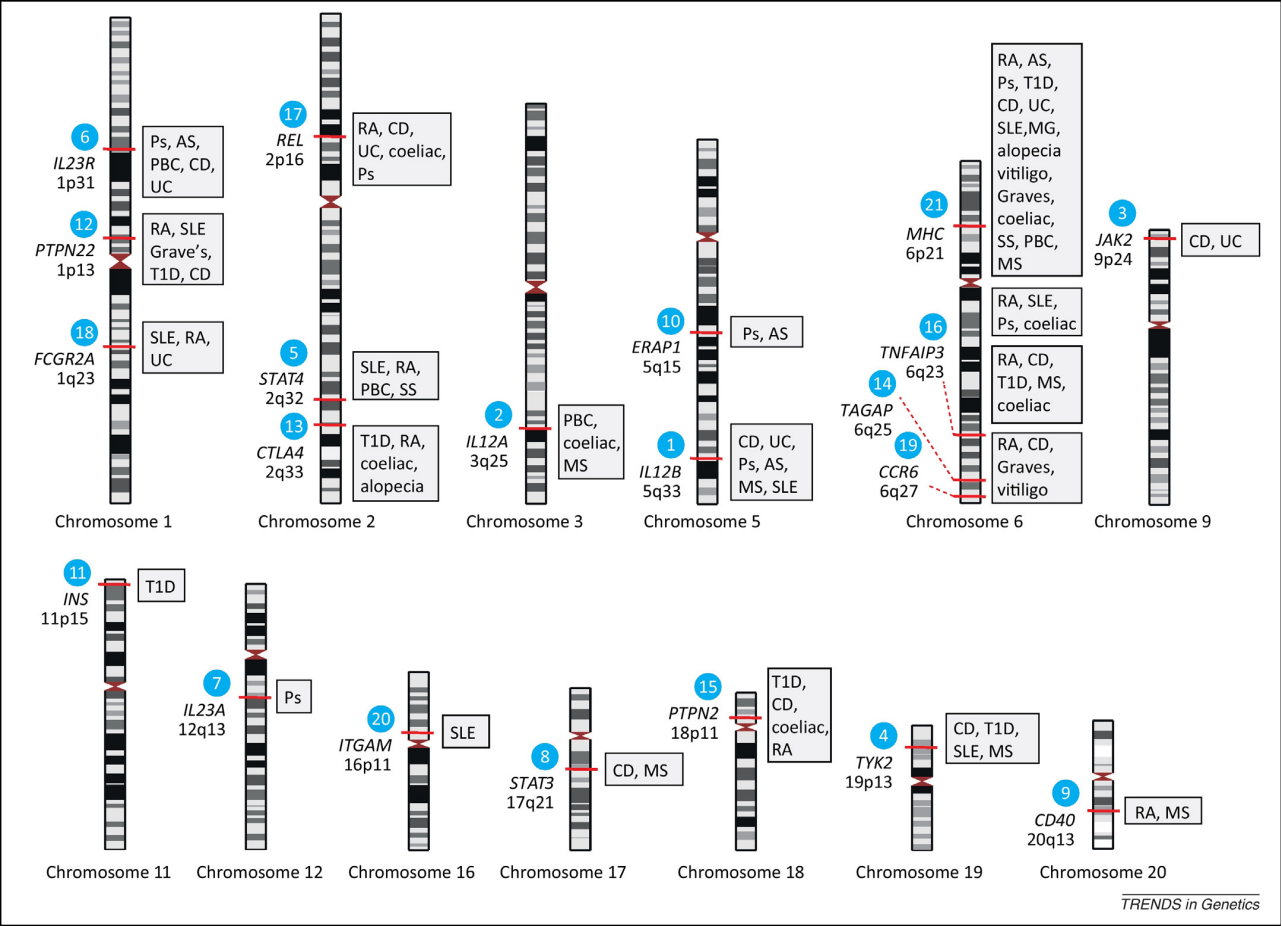
Examples of common autoimmune diseases where loci have been reported by GWAS. Blue filled numbered circles correspond to those shown in the overview of the immune system (Figure 1). This is not an exhaustive list and illustrates the overlap seen at many associated loci for different autoimmune diseases and how genes encoding different components of pathways may be associated with a given disease using data from [6] and the National Human Genome Research Institute (NHGRI) Catalogue of Published Genome-Wide Association Studies (www.genome.gov/gwastudies/) where full details can be found. It should also be noted that, in the majority of cases, the genes listed are candidates based on the genomic locus that was associated, and causality has not been established except in a small number of instances. Abbreviations: alopecia, alopecia areata; AS, ankylosing spondylitis; CD, Crohn's disease; coeliac, coeliac disease; Graves, Grave's disease; MG, myasthenia gravis; MS, multiple sclerosis; PBC, primary biliary cirrhosis; Ps, psoriasis; RA, rheumatoid arthritis; SLE, systemic lupus erythematosus; SS, systemic sclerosis; T1D, type 1 diabetes; UC, ulcerative colitis.

Genomic approaches such as GWAS have allowed previously unrecognised genomic loci, genes, proteins, and pathways involved in a particular process to be identified. In this context GWAS have been a notable success, particularly in terms of autoimmune and other immune-related conditions (Box 2, Figures 1 and 3), as exemplified by studies discussed here that highlight not only novel insights into disease pathogenesis but also the previously unappreciated extent of overlap between diseases in terms of associated loci [6,7]. However, considerable challenges remain: although GWAS have provided a genomic route-map of likely functional loci, in most instances we do not know what the specific causal functional variants are, which genes are being modulated, and how they relate to disease.

Another layer of complexity is added by the fact that, in most instances, non-coding sequence variants have been causally implicated by GWAS, and immune dysfunction is no exception to this trend. In these cases, expression quantitative-trait mapping offers an important complementary approach for resolving regulatory genetic variants and the genes affected. Interestingly, these studies have revealed significant overlap between disease and expression-associated genetic markers [8–10], and have pointed to commonalities between diseases historically considered unrelated. The extent of sequence level and structural genomic diversity revealed by sequencing studies such as the 1000 Genomes Project [11], combined with the extraordinary complexity of the genomic regulatory landscape being revealed by advances in functional genomics such as the ENCODE Project [12,13], highlight the challenges and opportunities that lie ahead in identifying functionally important genetic variation in non-coding regions and its consequences for immune function.

In addition to understanding genetic variation in both coding and regulatory regions, progress in understanding disease pathogenesis and opportunities for treatment of immune diseases will require the integration of genetic and environmental risk in a much more holistic way than has been achieved to date, taking into account epigenetic processes and context-specific effects in immune function [12,14,15]. In this review I highlight some of the insights gained from applying genomic approaches to Mendelian immune diseases and to polygenic autoimmune disorders, explore the role of regulatory and structural variants in the MHC and genome-wide, and suggest important directions for future research.

## Application of genomic approaches to primary immunodeficiency disorders

Historically, genetic investigation of rare Mendelian primary immunodeficiency disorders has been highly informative in understanding immune function [16], and examples are illustrated in Figures 1 and 2. Such ‘experiments of nature’ [17] proved in many cases to be tractable to classical linkage analysis and association mapping. Characterised traits range from severe combined immunodeficiency syndromes, with specific defects in cellular and humoral immunity, to defects in innate immunity such as impaired TLR signalling (Box 1, Figure 2). Elucidation of the bare lymphocyte syndromes for example was extremely important in resolving mechanisms of immune regulation, notably the nature and role of the HLA class II master regulator CIITA [18] and the RFX complex in regulating class II gene promoters [19].

Primary immunodeficiencies characterised by significant autoimmune dysfunction have provided important insights into central tolerance from study of the rare disease autoimmune polyendocrine syndrome 1 (APS1) and the identification of mutations in the autoimmune regulator (AIRE), a transcription factor regulating expression of self antigens in the thymus [20]; and of peripheral tolerance through definition of the role of the transcription factor FOXP3, mutation of which causes IPEX (immune dysregulation polyendocrinopath enteropathy X-linked) and results in autoimmunity through dysfunction of regulatory T cells [21,22]. Rare single-gene defects modulating B cell function and antibody production that result in immunodeficiency have been similarly informative (Figure 2). Examples range from investigation of X-linked agammaglobulinaemia, revealing the essential role for the tyrosine kinase BTK in B cell receptor signalling and maturation, to mutations underlying X-linked hyper IgM syndrome demonstrating that CD40 is essential for class switching and development of B cell memory [2].

Despite these successes, the identification of causative genes for some primary immunodeficiencies through classical approaches has been hampered by the small numbers of informative cases. Recent developments in genomic technology such as whole-exome sequencing (WES) have opened the door to resolving these rare instances and promise to advance this field significantly. This approach has highlighted how both loss-of-function and gain-of-function alleles of STAT1 (a key transcription factor involved in IFN signalling and susceptibility to mycobacterial and viral infection) may perturb normal immune function, leading to clear disease phenotypes [17], in this case, chronic mucocutaneous candidiasis (CMC), a persistent or recurrent infection with *Candida albicans* involving the nails, skin, oral, or genital mucosa [23]. Although a variety of genetic aetiologies have been identified, here the cause of autosomal dominant CMC was specifically investigated. The strategic approach of using WES to analyse a small number of individuals and then sequence the coding region of the implicated gene in a larger number of individuals was highly productive. Overall, 36 patients from 20 kindreds were found to be heterozygous for one of 12 identified mutations involving the coiled-coil domain of STAT1 [23]. The work also illustrates a significant advantage of studying immune traits, namely that functional characterisation is relatively tractable given that most of the relevant cell types and tissues are accessible. In the STAT1 study a clear role for the identified gain-of-function alleles was found in terms of impaired IL-17 immunity [23].

Although the cost of WES and whole-genome sequencing is decreasing, it remains a less common genomic approach than GWAS. The rarity of primary immunodeficiency disorders makes it challenging, and in some instances impossible, to identify sufficient cases for the GWAS to be adequately powered because this approach requires large numbers of cases and controls to test for association using a genome-wide panel of genetic markers. Nevertheless, for the most common immune-deficiency disorders, common variable immunodeficiency (CVID) and selective IgA deficiency, such studies have recently been performed. In the case of CVID, a GWAS involving 363 patients confirmed the role of the MHC, but also implicated other loci such as the disintegrin and metalloprotease (ADAM) genes as well as many rare structural variants (insertions and deletions) involving for example *ORC4L*, a gene important for initiation of DNA replication that was previously implicated in B cell lymphoproliferative disorders [24]. The genetic basis of CVID remains unresolved [2], but it is hoped that through improved clinical phenotyping [25] and genomic approaches the currently heterogeneous syndrome of CVID may be resolved into distinct disorders which will aid in the development of more targeted therapies and improved clinical care.

A recent similarly sized GWAS investigating IgA deficiency also confirmed the previous MHC class II association with this disease [26]. Subsequent fine mapping and imputation of HLA type resolved the primary association signal to HLA-DQB1*02 [27], which is also associated with type 1 diabetes (T1D) and coeliac disease. Intriguingly, the GWAS demonstrated evidence of an additional non-MHC association involving two loci previously implicated in autoimmune disease [26]. These included *IFIH1*, which encodes an interferon-inducible RNA helicase important in response to viral infections and implicated by GWAS in psoriasis [28] and in T1D [29]. Interestingly, other rare variants of *IFIH1* were also subsequently found to show independent association to T1D [30]). Similarly, the second locus, *CLEC16A*, which encodes a member of the C-type lectin domain family, is also associated with T1D, multiple sclerosis (MS), systemic lupus erythematosus, and rheumatoid arthritis (RA) [31], suggesting some overlap in disease pathogenesis [26].

## GWAS generate overlapping roadmaps for understanding autoimmunity

The associated loci shared between IgA deficiency and other immune diseases reflects a general trend that has been uncovered through GWAS, particularly of autoimmune diseases (Box 2), namely the overlap between the pathways and mechanisms involved in immune-related diseases [32]. In a recent analysis, among 107 independent non-MHC single nucleotide polymorphism (SNP) markers associated at genome-wide significance across seven common autoimmune diseases, 44% associated with at least two diseases [7]. Intriguingly, in 8% of instances the effects were shared but in an opposite direction, with increased risk in some diseases and protection in others. This is seen for example with *PTPN22* R620W; it is associated with increased risk of RA, thyroid disease and T1D, protection for Crohn's disease, and no effect on MS [6]. Sharing was also notable in associations involving cytokine signalling, pathways involved in B and T cell activation, innate immunity, and response to pathogens [6]. These shared patterns may provide insights into common mechanisms and unexpected overlaps in disease pathogenesis.

One outcome of identifying these shared associations is the potential to treat diseases with drugs previously approved for seemingly unrelated diseases (drug repositioning). Indeed, it has been found that genes currently implicated by GWAS are significantly more likely to be ‘druggable’ by small molecules (21%), or potentially modulated by ‘biopharmaceuticals’ (therapeutic antibodies/protein therapeutics) (47%), than those derived from considering the whole genome (17% and 38% respectively) [33]. One example of a candidate drug that could be repurposed for Crohn's disease is an existing monoclonal antibody against TNFSF11 (currently used for treating osteoporosis and bone cancer) based on the reported GWAS signal for that disease with *TNFRSF11* [34].

## The MHC: still challenging 40 years on

When considering the role of genetic variation in the immune system, the highly polymorphic MHC locus on chromosome 6p21 is sometimes viewed as being too complex and difficult to resolve, but this region of the genome is undoubtedly the elephant in the room that refuses to go away. GWAS have underlined what was already known from associations based on serological testing in the early 1970s, namely that the predominant genetic risk for autoimmune disease resides in the MHC, together with associations for infectious and inflammatory diseases as well as traits such as cancer and drug hypersensitivity [35]. It remains most likely that current autoimmune-disease risk alleles reflect past selective pressures on populations exposed to an environment rich in severe infectious diseases [35]. Infectious disease is important in driving polymorphisms [36], and there is evidence of positive selection involving coding variants altering the peptide binding grooves of MHC molecules. Selection is also evident in non-MHC GWAS loci such as *IL12A*, *IL18RAP*, and *SH2B3* in the context of coeliac disease [37]. The extraordinary degree of polymorphism in the MHC and extensive linkage disequilibrium have made fine-mapping of disease associations challenging [35,38]. Indeed current genomic technologies such as whole-genome sequencing and RNA sequencing are currently of limited utility in the MHC due to difficulties such as read mapping bias and reliability of variant calling. However, these difficulties should be alleviated as sequencing read lengths increase as well as through *de novo* sequence assembly [39]. A major success in applying genomic methods to understanding the MHC is SNP genotyping to establish HLA type [40,41]. This has been recently shown to be highly accurate for imputing HLA type to four-digit resolution for most classical alleles [40], and may replace conventional serological testing.

The majority of autoimmune-disease associations are thought to arise completely or in part from structural variants involving HLA class I and II molecules, acting in conjunction with a variety of known and unknown germline and somatic genetic variants, epigenetic mechanisms, and specific environmental triggers to modulate tolerance (for example resulting in a permissive environment for T cells recognising self antigens [42]). The combination of these factors cumulatively results in a specific disease phenotype. A recent study investigating structural variants in patients with RA demonstrated the power of using multiple large GWAS cohorts, imputation of HLA type, and sophisticated conditional analysis [43]. This study significantly advanced the longstanding shared-epitope hypothesis for susceptibility to RA involving consensus amino acid sequences spanning positions 70–74 in the β1 subunit of HLA-DR, which were implicated but could not fully explain the disease association. Analysis of six GWAS cohorts involving 19 992 individuals, including 5018 cases of anti-citrullinated peptide-positive RA, at the level of SNP, haplotype, and HLA type revealed the strongest association was with rs17878703, corresponding to amino acid position 11 of DRβ1, with further independent signals revealed for variants at amino acids 71 and 74, all located centrally in the peptide-binding groove [43]. Serial conditional analysis revealed further independent effects for amino acid position 9 in HLA-B and in HLA-DPβ1. This elegant work is undoubtedly a further significant advance but will require careful functional characterisation to establish causality and a clear understanding of mechanism.

Recent GWAS have also underlined the role of interactions involving the MHC, for example of HLA-B27 and genetic variation in *ERAP1* [44], a gene encoding an endoplasmic reticulum aminopeptidase involved in peptide trimming before HLA class I presentation. The situation is complex, with other new functional evidence linking pathogenic HLA-B27 homodimers with binding to natural killer and related immunoreceptors such as KIR3DL2 [45]. KIR receptors are mainly expressed on natural killer cells and are critical to maintaining tolerance, binding HLA class I ligands on target cells. Indeed the KIR gene locus is, similarly to the MHC, extremely polymorphic. For infections such as HIV-1, analysis of both MHC and KIR diversity has proven highly informative, notably for *HLA-B* alleles and variants involving *KIR3DL1* and *KIR3DS1*, inhibitory and activating receptors respectively, and recent work also implicates copy-number variation [46]. The KIR locus is currently not well captured by GWAS genotyping arrays but is the subject of active research interest.

MHC interactions are also being highlighted by recent expression quantitative-trait mapping studies in which possession of specific genetic variants in this region show association with differential expression of genes elsewhere in the genome. These include a reported GWAS SNP for ulcerative colitis in the MHC class II region associated with expression of *AOAH*, a gene encoding the enzyme acyloxyacyl hydrolase which plays a key role in the inflammatory response to Gram-negative bacteria located on chromosome 7p14 [47]. This association was shown to be cell type-specific and resolved to HLA class II alleles HLA-DRB1*04, HLA-DRB1*07, and HLA-DRB1*09 [48]. Intriguingly, these alleles associated with reduced *AOAH* expression were found to have a second monocyte-specific *trans* association, in this case to higher expression of *ARHGAP24*, which encodes a negative regulator of Rho GTPase-activating protein, suggesting new candidates to investigate when considering the known autoimmune-disease associations of these alleles [49].

The hypothesis that regulatory variants play a role in the many observed disease associations for the MHC is consistent with evidence from characterisation of specific loci, such as *HLA-C*, where local association was found with differential gene expression that involved a genetic variant associated with HIV-1 control [50]. Recent work suggests this association may arise from a linked variant in the 3′-UTR and differential binding of a microRNA [51]. More generally, expression quantitative-trait mapping has demonstrated many strong associations with this region of the genome [38]. Care is needed, however, because the polymorphic nature inherent to the MHC risks confounding results through differential hybridization of probes on conventional microarray platforms in the presence of a variant allele. Recent work using a custom MHC array has interrogated gene expression using lymphoblastoid cell lines homozygous for specific disease risk haplotypes [52]. This array included alternative allele probes at the site of a given single-nucleotide variant together with a tiling path design able to quantify expression specific to both sense and antisense strands that included intergenic regions and for the class III region as well as known and predicted splice junctions. This revealed that haplotype-specific transcription was common, comprising up to 11.1% of transcriptionally active regions and involving 96 genes [52]. Haplotypic differences were noted to often involve alternative splicing, which was significantly enriched in the MHC. Consistent with these results, expression quantitative-trait mapping resolved *cis*-acting associations. This work provides several candidate genes for further study to assess the contribution of regulatory variants to MHC disease associations.

## Expression quantitative-trait mapping and immune phenotypes

Expression quantitative-trait mapping in humans has been of growing interest following the recognition that gene expression is a heritable trait showing significant variation within and between populations that can be resolved based on linkage or, more recently, association to specific SNP markers [53]. Following the successful use of ‘genetical genomics’ in model organisms [54], this approach now provides a powerful tool for mapping regulatory genetic variants that is complimentary to, and can be highly informative for, GWAS for disease traits. One of the first studies adopting this approach studied lymphoblastoid cell lines, established from a cohort of children with asthma, which found that expression-associated SNPs (eSNPs) modulating *ORMDL3* were also the most significant GWAS SNPs for asthma [55]. Current work suggests the association with expression may be tissue specific [56].

In terms of immune phenotypes, recent expression quantitative-trait mapping studies have proved to be highly informative, notably for autoimmune diseases [48,57,58]. Analysis of primary immune cell types has demonstrated a high level of cellular specificity in eSNPs, underlining the importance of context specificity for regulatory variants. A recent study of paired samples of monocytes and B cells from healthy volunteers revealed the majority of associations were cell type-specific, notably among *trans*-associations [48]. Moreover, the analysis highlighted how directional effects occur for genes similarly expressed in both cell types in which an eSNP may show opposite directions of association, such as for the cell surface receptor CD62L important in the local inflammatory response [48]. Several cell type-specific *trans*-associations were resolved, including a B cell-specific association involving 12q13.2, a major autoimmune GWAS locus for several traits including T1D [48]. This showed that, in addition to the previously investigated and controversial local associations with expression of *RPS26* [59], *trans*-association is present for *IP6K2* and a transcript mapping 5′ to *CDKN1A*, which suggests a role for p53 mediated apoptosis and cell-cycle regulation. Overall, 49.4% of traits in the National Human Genome Research Institute (NHGRI) Catalog of Published Genome-Wide Association Studies (17.3% of reported GWAS SNPs) were associated with one or more eSNPs in this dataset, with 4.6% of GWAS SNPs showing association with expression of different genes in a cell type-specific manner [48]. GWAS hits for systemic lupus erythematosus, for example, mainly involved B cell-specific eSNPs, whereas for ulcerative colitis the associations were mainly monocyte-specific, consistent with the contrasting aetiologies of these two diseases involving adaptive and innate immunity.

Haematological traits relevant to immunity, such as white-cell count and abundance of specific cell subtypes, have a heritable component contributing to observed variation. Among individuals of African ancestry for example, lower white-cell counts are observed, and this was found to be associated with a specific null regulatory variant of *DARC*, a variant that confers a strong selective advantage in malaria [60]. Recent GWAS have resolved several associations [61,62], including variants at chromosome 17q21 near *CSF3* that show association with neutrophil and total white blood cell count [61,63,64]. This is interesting given the disease association of this locus with RA and asthma, and the clinical utility of granulocyte colony stimulating factor (encoded by *CSF3*) in treating neutropenia. Other striking associations (reviewed in [62]) include variants at chromosome 2q31 near *ITGA4* with monocyte count. *ITGA4* encodes a component of integrin important in white-cell tissue migration.

## Epigenetic variation, functional genomics, and the immune system

Epigenetic mechanisms modulating gene expression, such as DNA methylation, histone modifications, and non-coding RNAs, play a critical role in the immune system [15,65]. This is manifest in the complex and essential regulation of immune-cell identity and function during development and differentiation to generate multiple cell lineages, with remodelling of the epigenome acting to restrict or promote gene expression and determine cell fate (Box 3). Epigenetic regulation has been found to be highly dynamic and responsive, providing an important interface with the environment and memory of past exposures. Several environmental risk factors for autoimmune disease have been linked through epigenetics including cigarette smoking, infections such as Epstein–Barr virus, exposure to reproductive hormones, and vitamin D deficiency [66]. Gene–environment interactions have been resolved for example in RA between cigarette smoking and *HLA-DRB1* risk alleles [67] together with other gene loci such as *PTPN22* [68]. Epigenomic approaches can be informative for specific environmental risk factors such as vitamin D deficiency. The vitamin D receptor (VDR) is a ligand-activated transcription factor, and recent analysis of VDR genome-wide occupancy using ChIP-seq revealed that loci associated by GWAS in several autoimmune conditions including *IRF8* (associated with MS) and *PTPN2* (Crohn's disease and T1D) were bound by VDR [69]. For specific disease risk alleles in the MHC, there is evidence suggesting allele-specific recruitment of VDR at the promoter of *HLA-DRB1*, which illustrates how environmental and genetic risk factors may be related [70].

Genomic approaches such as GWAS and WES have proven very powerful in defining the extent and nature of genetic variation in the context of immune function and disease. However, considerable challenges remain, not least in establishing causal functional variants (Box 4). The design of functional studies requires careful consideration of context-specific effects, with increasing emphasis on analysis of primary cells in a disease setting where complementary functional genomic, epigenomic, and proteomic approaches can be applied together with immunological assays and testing of hypotheses in model organisms. Interpretation will be facilitated by adoption of a more systems-based approach and the analysis and integration of genetic, epigenetic, and environmental modulators of disease. Expression quantitative-trait mapping has proven a valuable tool to resolve regulatory variants, and future studies in specific contexts should be very helpful in defining the extent and nature of genetic associations, in particular those involving *trans*-acting variants. The use of RNA sequencing should further increase the informativeness of such studies, identifying alternatively spliced transcripts and non-coding RNAs as well as allele-specific expression involved in immune dysfunction.

## Concluding remarks

Genetic variation plays a fundamental role in modulating the development and effective functioning of the immune system. Recent work has illustrated how we can use genomic and epigenomic approaches to define such variation and understand the immune response in health and disease. This offers the promise of improved insights into disease pathogenesis, better diagnostic accuracy, new or repositioned therapeutics targeted to maximise benefit for the individual, and opportunities to improve outcome. To deliver on this will require continued excellence in basic science and translational research spanning genetics and immunology which takes advantage of current technological advances and our ability to resolve ‘experiments of nature’ seen in immune dysfunction.

## Box 1 Overview of aspects of immune system function and disease

The immune response can be traditionally divided into innate and adaptive immunity (Figure 1), although overlap exists. The evolutionarily ancient innate immune response provides a very rapid defence mechanism (within minutes of infection) involving inflammation, complement activation, phagocytosis, and destruction of pathogens. The innate immune response is critically dependent on pattern-recognition receptors (PRRs), such as Toll-like receptors (TLRs) found on the cell surface or endosomes in effector cells (including macrophages, neutrophils, and dendritic cells), that recognise pathogen-associated molecular patterns (PAMPs) typically located on the surface of pathogen cells. The resulting gene activation leads to cytokine and chemokine release and generation of an inflammatory response.

By contrast, the adaptive immune response typically takes days to become effective following first exposure, and involves B and T lymphocytes with recognition of antigens and the generation of a specific antibody-mediated (humoral) response together with cell-mediated immunity involving, for example, T helper cells and cytotoxic T cells. The humoral response will eliminate pathogens and allows for generation of immunological memory. Antibodies may act, for example, to neutralise bacterial toxins, opsonise bacteria to target them (for example promoting phagocytosis), or result in complement activation.

Antigens are presented by specific molecules encoded by the major histocompatibility complex (MHC) on chromosome 6p21 and involves specialised antigen-presenting cells including dendritic cells, monocytes, and B cells. This may involve the endogenous pathway (for example in viral infection), in which the peptide is loaded onto MHC class I molecules and presented to CD8^+^ cytotoxic T cells (Figure 1), or the exogenous pathway (bacteria, parasites) and loading onto an MHC class II molecule and presentation to a CD4^+^ T helper cell. The MHC is a highly gene-dense and polymorphic region which includes the classical human leukocyte antigen (HLA) class I and class II genes and shows remarkably strong disease associations with a broad range of autoimmune, infectious, and inflammatory diseases.

Autoimmune diseases involve a failure of self-tolerance and an immune response to a self-antigen with the presence of self-reactive CD4^+^ T lymphocytes and typically autoantibodies. Autoimmune diseases are often familial and include a range of common devastating conditions that may be organ-specific (e.g., type I diabetes, multiple sclerosis, autoimmune thyroid disease, coeliac disease, Crohn's disease, ulcerative colitis, psoriasis, rheumatoid arthritis) or systemic (e.g., systemic lupus erythematosus, dermatomyositis, scleroderma).

## Box 2 Autoimmune disorders and GWAS

GWAS have been highly informative in autoimmune diseases, uncovering associations with many different genomic loci beyond the MHC and providing new insights into pathogenesis (Figures 1 and 3). Over 200 genomic loci have been implicated in autoimmune disease [6]. These have been extensively reviewed [6,42,71], and a very useful source of current information about these data is the NHGRI Catalogue of Published Genome-Wide Association Studies (www.genome.gov/gwastudies/).

Autoimmune diseases such as Crohn's disease have been highlighted as significant GWAS success stories in which new pathways and potential drug targets have been resolved. This includes genes encoding components of cytokine signalling, notably *IL2RA* and *IL23R*, as well as of innate immunity including autophagy (for example *ATG16L1*) and *NOD2* (which acts as a sensor of bacterial peptidoglycan), although the role of *NOD2* was identified prior to GWAS [72]. It is striking that genetic variation involving genes at diverse genomic locations, but encoding proteins involved in a given pathway, should be associated with a particular immune phenotype or related phenotypes; for example, strong associations have been recognised between IL-23/IL-12 signalling pathways and several autoimmune diseases including inflammatory bowel disease, psoriasis, and primary biliary cirrhosis [6,73] (Figures 1 and 3). In some instances, such as for *PTPN22* (which was initially identified by a candidate gene approach [74]), the functional risk allele has been established – in this case a nonsynonymous variant (rs2476601, R620W) disrupting binding between the intracellular phosphatase encoded by *PTPN22* and the kinase Csk, altering T and B cell responsiveness to receptor stimulation. In the vast majority of cases the modulated gene and functional variant remains unresolved and is likely in many instances to involve functional regulatory elements in non-coding DNA [4,12,75].

The human microbiome (microbes that live in and on our bodies) is increasingly recognised as a critical modulator of the host immune system and is the subject of intensive research [76]. The role of genetic variation in this relationship is likely to be highly significant, as illustrated by recent work involving a GWAS locus for rheumatoid arthritis, ulcerative colitis, and coeliac disease at *TNFRSF14* (*HVEM*) [77]. The gene was found to encode a member of the tumor necrosis factor receptor superfamily that is an important orchestrator of mucosal immunity and defence against pathogenic bacteria [77].

A recent example of how GWAS signals may be informative for understanding disease pathogenesis and use of therapeutics comes from work on a highly significant GWAS signal for multiple sclerosis, localising to *TNFRSF1A* with a modest magnitude of effect [odds ratio for risk allele = 1.12 (1.11–1.14)]; further study revealed a functional association with the production of a novel stable soluble form of the encoded TNF receptor 1 (TNFR1) [78]. This variant occurs when the risk allele is alternatively spliced to produce a transcript lacking exon 6 and a protein capable of antagonizing TNF. This is consistent with the worsening of multiple sclerosis seen with anti-TNF treatment, which is in contrast to other autoimmune diseases, and it was striking that this SNP has not been associated with other autoimmune conditions.

## Box 3 Epigenetics and immune cell development, differentiation, and function

Dynamic epigenetic marks are critical in determining whether CD4^+^ T helper cells differentiate down a Th1 or Th2 pathway on leaving the thymus, as seen for example by gain of active or repressive histone modifications/DNA methylation at the IFNγ and Th2 cytokine gene locus [65]. In terms of B cell differentiation, directional DNA methylation changes play an important role [79], as illustrated by changes in the promoter of the developmentally regulated gene *CD79A* [80]. Epigenetic mechanisms are also critical to assembly of B and T cell receptors through V(D)J rearrangement, and changes in DNA methylation, histone modifications, chromatin accessibility, and structure are important in controlling accessibility of recombination signal sequences [81]. Epigenetics is also thought to be important in immunological memory, and recent evidence from work involving *Candida albicans* demonstrated the concept of training of the immune system through epigenetic modifications (in this case stable changes in histone trimethylation at H3K4), leading to protection against reinfection [82].

## Box 4 Challenges to using genomic approaches to understand immune function, and future directions

GWAS have achieved considerable success in resolving association with common traits, notably in the context of autoimmune disorders as described. There are of course caveats to such enthusiasm, notably in terms of the modest magnitude of effect and overall small proportion of estimated heritable risk explained, the challenges of moving from associated locus to causal functional variant, and indeed even implicating specific modulated genes based on associated SNP markers [12,83]. For whole-exome and whole-genome sequencing datasets in which rare variants are sought to resolve the basis of immune traits such as those showing Mendelian inheritance, the analysis of such datasets also remains challenging, and the tools available and optimal strategies for experimental design are incompletely developed [84]. Moreover, it is important to note that combining the use of technologies to resolve sequence and structural genomic variation with careful immunological studies of function to establish causal relationships remains paramount to successful implementation of current genomic approaches and the identification of causal functional variants. It is also important to note that our ability to study and quantify genomic variation is very much focused on DNA sequence variation, and we are currently not well-equipped to dissect either the more repetitive regions of the genome or a significant proportion of structural genomic variants. Ongoing and future research should provide new approaches to interrogate such regions and variants in the context of the immune system. Similarly, the technologies for epigenomic profiling, for example of DNA methylation at genome-wide resolution, are not yet sufficiently developed for widespread adoption. Finally, the often enigmatic nature of the MHC seems likely to continue to keep its secrets for some time more because the difficulties of applying high-throughput sequencing to this region of the genome remain very significant due to the high level of sequence and structural polymorphism found in this region.
